# Preparation and Antibacterial Performance Study of CeO_2_/g-C_3_N_4_ Nanocomposite Materials

**DOI:** 10.3390/molecules29235557

**Published:** 2024-11-25

**Authors:** Jingtao Zhang, Ruichun Nan, Tianzhu Liang, Yuheng Zhao, Xinxin Zhang, Mengzhen Zhu, Ruoyu Li, Xiaodong Sun, Yisong Chen, Bingkun Liu

**Affiliations:** 1 Key Laboratory of Vegetable Biology of Hainan Province, School of Food and Bioengineering, College of Tobacco Science and Engineering, Zhengzhou University of Light Industry, 136 Kexue Road, Zhengzhou 450002, China; 17862101323@163.com (R.N.); 13383782853@163.com (T.L.); zhaoyuheng0218@163.com (Y.Z.); 17621241225@163.com (X.Z.); 17344600322@163.com (M.Z.); 2 Key Laboratory of Vegetable Biology of Hainan Province, The Institute of Vegetables, Hainan Academy of Agricultural Sciences, 14 Xingdan Road, Haikou 571100, China; chenyisong@hnaas.org.cn; 3 School of Material and Chemical Engineering, Zhengzhou University of Light Industry, 136 Kexue Road, Zhengzhou 450002, China; 18568035226@163.com

**Keywords:** g-C_3_N_4_ nanocomposites, photo-Fenton reaction, antimicrobial activity, photocatalytic degradation, heterojunction

## Abstract

In response to the challenges of food spoilage and water pollution caused by pathogenic microorganisms, CeO_2_/g-C_3_N_4_ nanocomposites were synthesized via one-step calcination using thiourea and urea as precursors. Steady-state photoluminescence (PL) spectroscopy analysis demonstrated that 8 wt% CeO_2_/g-C_3_N_4_ exhibited superior electron–hole separation efficiency. Quantitative antimicrobial assays demonstrated that the nanocomposites displayed enhanced bactericidal activity against *Escherichia coli*, *Ralstonia solanacearum*, and *Staphylococcus aureus*. Electron paramagnetic resonance (EPR) spectroscopy analysis verified the generation of hydroxyl radicals (·OH) and superoxide radicals (·O_2_^−^) during the photo-Fenton process utilizing CeO_2_/g-C_3_N_4_ nanocomposites. Additionally, 8 wt% CeO_2_/g-C_3_N_4_ nanocomposites demonstrated enhanced photocatalytic degradation of rhodamine B (RhB) and tetracycline hydrochloride (TC) under photo-Fenton conditions.

## 1. Introduction

Pathogenic bacteria represent a critical threat to human health and well-being [1,2,3,4]. These microorganisms readily colonize common surfaces, contaminate potable water sources and food products, and disperse as airborne bioaerosols [5,6,7,8]. Pathogenic contamination is the primary cause of foodborne disease outbreaks, posing significant risks to global public health [9,10]. Conventional disinfection approaches include chlorination [11], ultraviolet irradiation [12], and ozonation [13]. However, these conventional methods exhibit considerable limitations, including substantial operational costs and the generation of harmful by-products [14,15,16]. Photocatalysis has emerged as a promising sterilization technology, offering distinct advantages, including absence of toxic by-products, economic viability, negligible microbial resistance development, and broad-spectrum antimicrobial activity [17,18,19].

Graphitic carbon nitride (g-C_3_N_4_), a metal-free polymeric semiconductor photocatalyst, demonstrates distinctive properties, including environmental benignity, high specific surface area, and abundant active sites [20,21,22,23]. Additionally, g-C_3_N_4_ serves as an effective support material for other photocatalysts, enhancing their photocatalytic activity [24,25,26]. Despite extensive investigation, the relatively low electron-hole separation efficiency of g-C_3_N_4_ remains a fundamental limitation for practical applications [27,28,29]. Strategies to address this limitation include elemental doping [30,31], defect engineering [32,33,34], and heterojunction construction [35,36,37]. Among these strategies, the construction of heterojunctions with complementary band structures has proven particularly effective in enhancing charge carrier separation efficiency [38].

Cerium oxide (CeO_2_), while extensively studied as a photocatalytic material, exhibits inherent limitations regarding visible light utilization and charge carrier recombination [39,40,41]. Zhao et al. [42] demonstrated that CeO_2_ and g-C_3_N_4_ exhibit complementary band structures conducive to S-scheme heterojunction formation, significantly enhancing photocatalytic activity and tetracycline degradation efficiency. Wang et al. [43] further confirmed that g-C_3_N_4_ incorporation substantially improves the photocatalytic performance of CeO_2_. Based on these findings, the CeO_2_/g-C_3_N_4_ heterojunction demonstrates potential for advanced photocatalytic applications.

In this study, CeO_2_/g-C_3_N_4_ nanocomposites were synthesized through a one-step calcination method using thiourea and urea as precursors. The structural, compositional, and optical properties of the synthesized materials were systematically characterized through X-ray diffraction (XRD), X-ray photoelectron spectroscopy (XPS), scanning electron microscopy (SEM), UV-visible spectroscopy, and photoluminescence (PL) analyses. The photo-Fenton antimicrobial efficacy was evaluated using *E. coli*, *R. solanacearum*, and *S. aureus* as model organisms. The investigations revealed that CeO_2_/g-C_3_N_4_ exhibited superior photo-Fenton disinfection efficiency, with complete inactivation of 2 × 10^7^ CFU/mL of *E. coli*, *R. solanacearum,* and *S. aureus* achieved within 30 min and 2 h, respectively. The photo-Fenton catalytic performance was assessed using rhodamine B (RhB) and tetracycline hydrochloride (TC) as target pollutants. The experimental results demonstrated that CeO_2_/g-C_3_N_4_ achieved degradation efficiencies of 97.2% and 83% for RhB and TC, respectively, with investigations conducted on the effects of catalyst loading, pH, and H_2_O_2_ concentration. Furthermore, mechanistic studies of the antimicrobial activity were performed to establish a fundamental basis for the practical application of g-C_3_N_4_-based photocatalysts.

## 2. Results

### 2.1. Material Characterization

**Figure 1:**
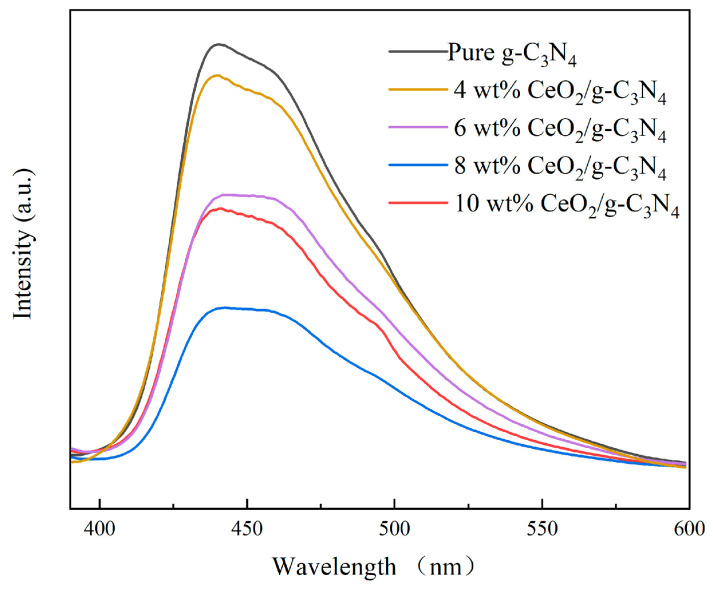
PL spectra of samples with different proportions.

The photocatalytic efficiency of g-C_3_N_4_ is fundamentally governed by its charge carrier separation capability [44]. Steady-state PL spectroscopy was utilized to evaluate the photocatalytic efficiency and optimize the compositional ratio of the synthesized materials [45]. As illustrated in Figure 1, the incorporation of CeO_2_ resulted in the substantial enhancement of the material’s photocatalytic performance. The PL emission spectrum of 4 wt% CeO_2_/g-C_3_N_4_ exhibited a peak intensity comparable to pristine g-C_3_N_4_, suggesting similar charge carrier separation characteristics. With increasing CeO_2_ content up to 8 wt%, the charge carrier separation efficiency demonstrated progressive enhancement. Rama et al. [46] attributed this enhancement to the optimized interfacial charge transfer dynamics within the heterojunction nanostructure. The optimal charge carrier separation efficiency was achieved with 8 wt% CeO_2_/g-C_3_N_4_ nanocomposites. Further increases in CeO_2_ loading beyond 8 wt% resulted in diminished charge carrier separation efficiency, likely due to excessive recombination centers.

**Figure 2:**
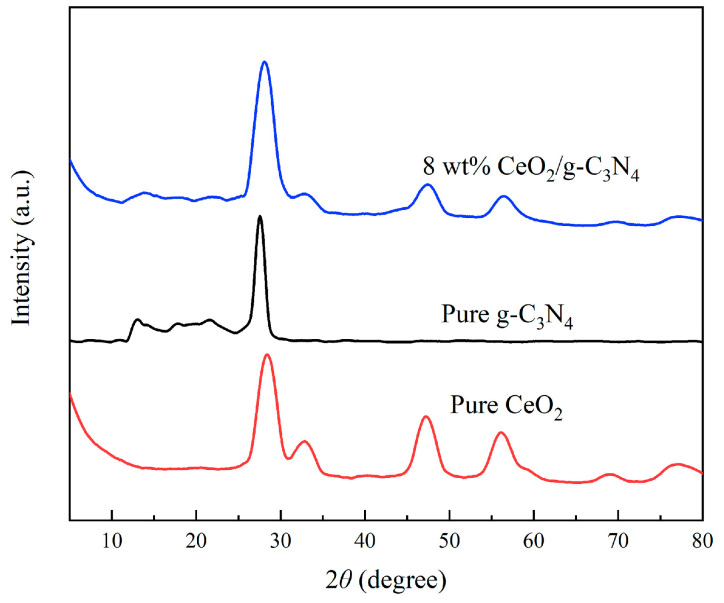
XRD patterns of different samples.

The crystallographic structure of the 8 wt% CeO_2_/g-C_3_N_4_ nanocomposite was characterized by XRD analysis, as illustrated in Figure 2. The diffraction pattern of pristine g-C_3_N_4_ exhibited a characteristic intense peak at 2*θ* = 27.4°, indexed to the (100) crystal plane. This distinctive diffraction peak (2*θ* = 27.4°) was attributed to the interplanar stacking of conjugated aromatic systems within the g-C_3_N_4_ structure [47]. The composite material exhibited a broad diffraction peak centered at approximately 28°, which represents the overlapping contributions from the CeO_2_ (111) plane at 28.5° and the g-C_3_N_4_ (110) plane at 27.4°. The observed diffraction patterns confirm the successful formation of a binary phase system in the CeO_2_/g-C_3_N_4_ nanocomposite, demonstrating the effective integration of both components while maintaining their respective crystalline structures.

**Figure 3:**
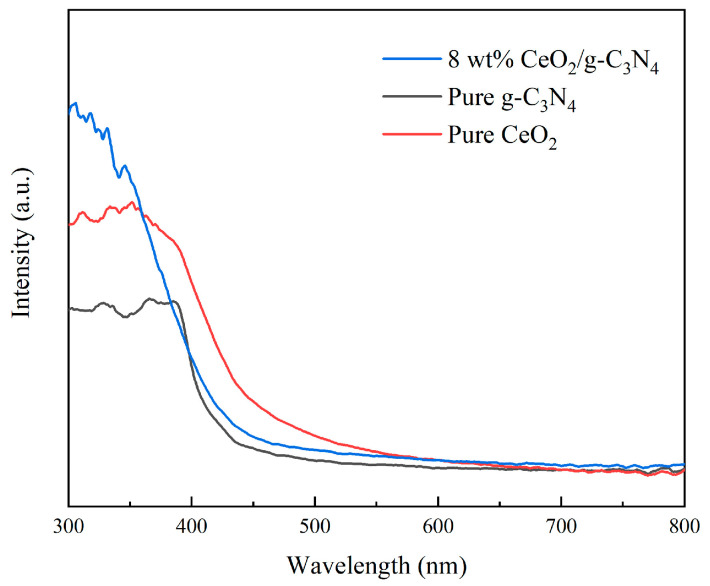
UV-vis spectra of different samples.

The optical properties of 8 wt% CeO_2_/g-C_3_N_4_ nanocomposites, pristine CeO_2_, and g-C_3_N_4_ were investigated using UV-vis absorption spectroscopy (Figure 3). Comparative analysis revealed enhanced absorption intensity in the UV region (250–400 nm) for the CeO_2_/g-C_3_N_4_ nanocomposite relative to pristine g-C_3_N_4_. Additionally, intensified absorption bands in the 250–300 nm region, compared to pristine CeO_2_, indicated a synergistic enhancement of optical absorption through heterojunction formation [48].

**Figure 4:**
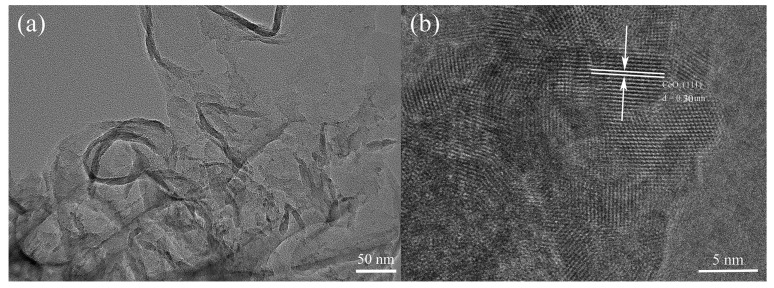
TEM (**a**) and HRTEM (**b**) spectrum of the 8 wt% CeO_2_/g-C_3_N_4_ heterojunction.

HRTEM was employed to elucidate the morphological characteristics, nanostructural features, and spatial distribution of the synthesized CeO_2_/g-C_3_N_4_ nanocomposites. The HRTEM micrograph (Figure 4a) revealed a characteristic layered morphology of g-C_3_N_4_ with inconsistently distributed CeO_2_ nanoparticles anchored on its surface. An analysis of lattice fringes (Figure 4b) revealed an interplanar spacing of 0.16 nm, corresponding to the (311) crystallographic plane of CeO_2_, corroborating the XRD findings.

**Figure 5:**
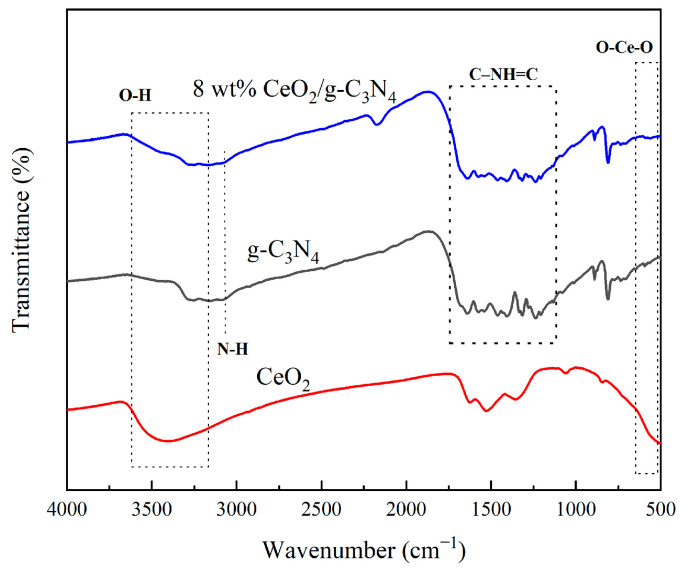
FTIR spectra of different samples.

The chemical composition and molecular interactions within the nanocomposites were investigated using FTIR spectroscopy. The broad absorption band centered at 3475 cm^−1^ was attributed to O−H stretching vibrations of surface-adsorbed water molecules, as previously reported by Gomathi et al. [49]. The characteristic absorption bands in the 1200–1750 cm^−1^ region corresponded to C−NH=C stretching vibrations within the triazine units of g-C_3_N_4_ [50]. The absorption band in the 3000–3500 cm^−1^ region was assigned to N−H stretching vibrations of primary and secondary amino groups. The peak observed in the range of 500–550 cm^−1^ was attributed to O−Ce−O vibrational modes, consistent with previous findings by Hu et al. [51]. Notably, all characteristic vibrational modes of both g-C_3_N_4_ and CeO_2_ were preserved in the 8 wt% g-C_3_N_4_/CeO_2_ nanocomposite, indicating the successful formation of the heterojunction structure, with significant interfacial interactions between the two components (Figure 5). 

**Figure 6:**
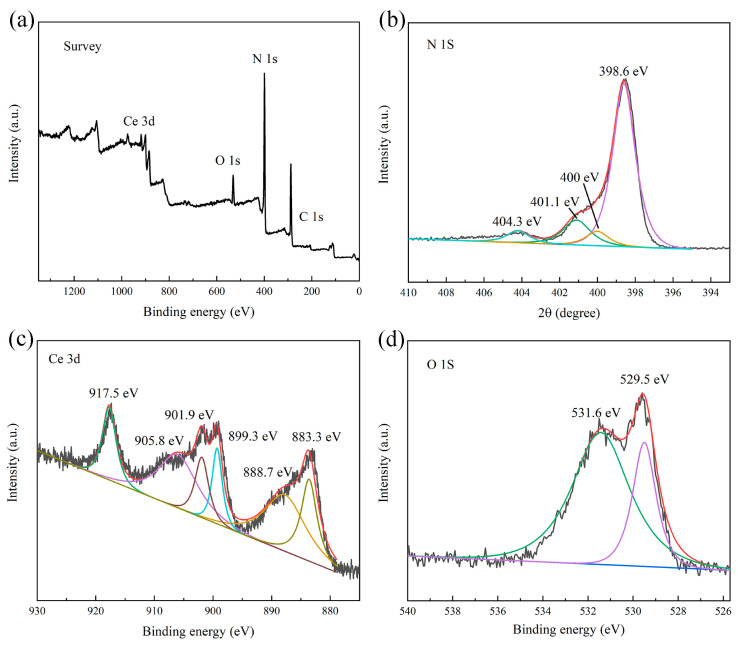
XPS spectra of 8 wt% CeO_2_/g-C_3_N_4_ nanocomposites. (**a**) Survey, (**b**) N 1s, (**c**) Ce 3d, and (**d**) O 1s.

XPS analysis was performed to elucidate the elemental composition and chemical states of the synthesized materials. The survey spectrum (Figure 6a) confirmed the presence of C, N, Ce, and O within the nanocomposite structure. Figure 6b shows the high-resolution spectrum of the N 1s orbital, revealing binding energies at 398.6 eV, 401.1 eV, and 404.3 eV, attributed to the sp^2^ orbital of C−N=C double bonds, π-bond excited C−N, and (C)_3_−N species, respectively [52,53]. Figure 6c presents the high-resolution spectrum of the Ce 3d orbital, where binding energies at 899.3 eV, 901.9 eV, 905.8 eV, and 917.5 eV were assigned to Ce^4+^ 3d_3/2_, Ce^3+^ 3d_3/2_, Ce^4+^ 3d_3/2_, and Ce^4+^ 3d_3/2_, respectively. The peak at 883.3 eV was attributed to Ce^4+^ 3d_5/2_ and the peak at 888.7 eV to Ce^3+^ 3d_5/2_ [54,55]. From the spectra, it can be seen that Ce existed mainly in the +4 valent and +3 valent forms. It was the main manifestation of photo-Fenton activity [55]. Figure 6d displays the high-resolution spectrum of the O 1s orbital, exhibiting main peak positions at 529.5 eV and 531.6 eV, characteristic of Ce−O and C−O bonds, respectively [56,57].

**Figure 7:**
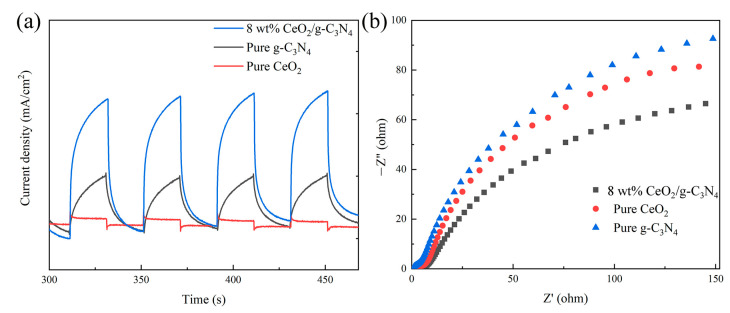
Different samples of (**a**) I-T and (**b**) EIS curves.

Photoelectrochemical properties were evaluated through transient photocurrent response (I-T) measurements and electrochemical impedance spectroscopy (EIS), as shown in Figure 7. The nanocomposite exhibited substantially enhanced photocurrent density, compared to its individual components (Figure 7a). The 8 wt% CeO_2_/g-C_3_N_4_ nanocomposite displayed decreased charge transfer resistance relative to pristine CeO_2_ and g-C_3_N_4_ (Figure 7b). These findings complement the PL spectroscopy results, indicating enhanced charge carrier separation efficiency and consequent improvement in photocurrent density [58]. Wang et al. [43] demonstrated that elevated Ce^4+^ content in CeO_2_/g-C_3_N_4_ photocatalysts facilitates photogenerated electron capture, promoting efficient charge carrier separation. These mechanistic insights corroborate our observed enhancement in charge carrier separation efficiency.

### 2.2. Fenton-like and Photocatalytic Sterilization Performance

**Figure 8:**
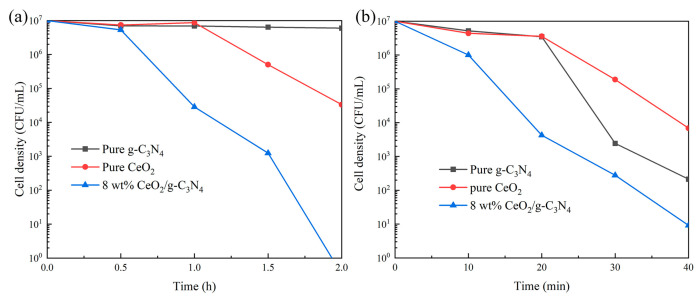
(**a**) Fenton-like sterilization curves of different samples. (**b**) Photocatalytic sterilization curve; (species of bacteria: *E. coli*; H_2_O_2_: 5 mM; light intensity: 30 mW/cm^2^).

The antimicrobial efficacy of the nanocomposites was evaluated under both Fenton-like and photocatalytic conditions (Figure 8). Under Fenton conditions with 5 mM H_2_O_2_ (Figure 8a), CeO_2_ demonstrated Fenton-mediated bactericidal activity, achieving a 2-log reduction in the bacterial population within 2 h, while g-C_3_N_4_ exhibited minimal activity. Notably, the nanocomposite demonstrated superior antimicrobial performance, achieving a 7-log reduction in the bacterial population within 2 h. Photocatalytic sterilization experiments (Figure 8b) revealed significant antimicrobial activity for both CeO_2_ and g-C_3_N_4_, achieving 3-log and 5-log reductions in the bacterial population within 40 min, respectively. The nanocomposite exhibited enhanced performance, achieving a 6-log reduction in the *E. coli* population within 40 min. The concurrent operation of Fenton-like and photocatalytic mechanisms produced a synergistic antimicrobial effect, resulting in superior bactericidal performance.

### 2.3. Photo-Fenton Performance Tests

**Figure 9:**
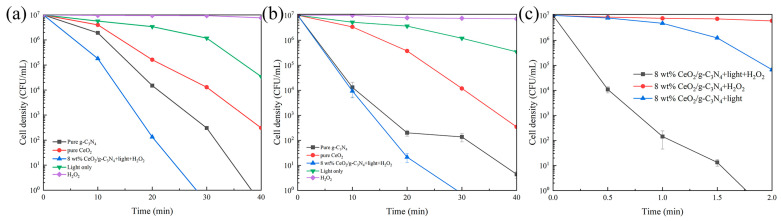
Photo-Fenton (**a**) *E. coli* curve; (**b**) *R. solanacearum;* and (**c**) *S. aureus* (H_2_O_2_: 5 mM; light intensity: 30 mW/cm^2^).

As shown in Figure 9a, both CeO_2_ and g-C_3_N_4_ demonstrated enhanced bactericidal efficiency under light irradiation, with g-C_3_N_4_ showing a marked improvement, despite its lack of intrinsic Fenton-like activity. Huang et al. [59] developed 0D/2D CeO_2_/g-C_3_N_4_ heterojunction photocatalysts, which achieved complete bacterial inactivation within 3 h, demonstrating efficient photocatalytic bactericidal performance. In comparison, the 8 wt% CeO_2_/g-C_3_N_4_ nanocomposite synthesized in this study achieved complete *E. coli* inactivation (initial concentration: 2 × 10^7^ CFU/mL) within 30 min under photo-Fenton conditions (Figure 9a). Figure 9b,c demonstrate that *R. solanacearum* and *S. aureus* (initial concentration 10^7^ CFU/mL) were completely inactivated within 30 min and 2 h, respectively. Under dark conditions, minimal antimicrobial activity was observed. These results indicate that the incorporation of CeO_2_ into g-C_3_N_4_ enhances reactive oxygen species (ROS) generation and promotes catalyst–microorganism interactions [60].

**Figure 10:**
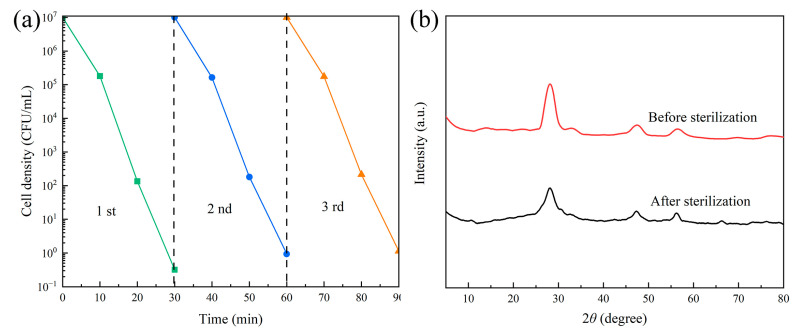
The 8 wt%CeO_2_/g-C_3_N_4_ nanomaterials of the (**a**) photo-Fenton reaction cycle experiment; (**b**) comparison of XRD spectra before and after (experimental conditions: *R. solanacearum* as model bacteria, [H_2_O_2_] = 5 mM, light intensity = 30 mW/cm^2^).

To assess the stability and reusability of the 8 wt% CeO_2_/g-C_3_N_4_ catalyst, cyclic photo-Fenton experiments were conducted over three consecutive runs, using *R. solanacearum* (2 × 10^7^ CFU/mL) as the target microorganism. As illustrated in Figure 10a, the 8 wt% CeO_2_/g-C_3_N_4_ catalyst maintained its bactericidal efficacy through multiple cycles, consistently achieving a seven-order magnitude reduction in *R. solanacearum* concentration, demonstrating excellent reusability. As can be seen in the XRD spectra before and after cycling in Figure 10b, the crystallinity of the composite catalyst slightly decreased after cycling, indicating the good catalytic stability of the catalyst.

### 2.4. Photo-Fenton Degradation of Pollutants Test

**Figure 11:**
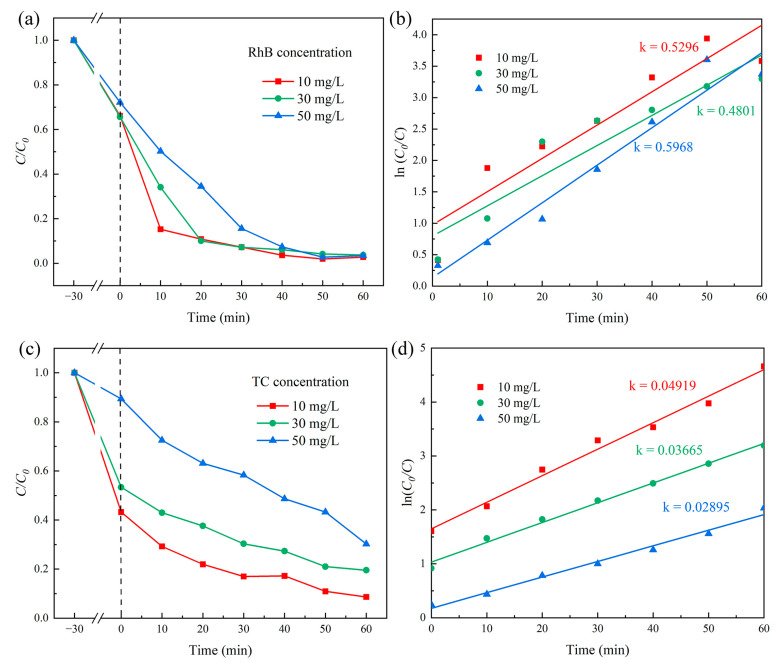
Photo-Fenton degradation of 8 wt% CeO_2_/g-C_3_N_4_ samples; (**a**) RhB and (**b**) kinetic curves; (**c**) TC and (**d**) kinetic curves (amount of catalyst: 20 mg; H_2_O_2_: 5 mM; light intensity: 100 mW/cm^2^).

RhB and TC were employed as model pollutants to investigate the degradation performance under photo-Fenton conditions at varying concentrations. The experiments were performed using initial pollutant concentrations of 10.0, 30.0, and 50.0 mg/L, with a catalyst loading of 15 mg and H_2_O_2_ concentration of 5 mM. Figure 11a illustrates the photo-Fenton degradation kinetics of RhB using the 8 wt% CeO_2_/g-C_3_N_4_4 sample. Under dark conditions, RhB exhibited adsorption capacities of 33.9%, 34.4%, and 27.9% for the respective initial concentrations. Following 60 min of photo-Fenton reaction, the degradation efficiencies reached 97.2%, 96.5%, and 96.3%, respectively, with corresponding reaction rate constants (Figure 11b) of 0.05296 min^−1^, 0.04801 min^−1^, and 0.05968 min^−1^. The 8 wt% CeO_2_/C_3_N_4_ nanocomposite demonstrated an efficient photo-Fenton degradation of RhB, achieving 96.3% degradation, even at an elevated concentration of 50 mg/L. Figure 11c presents the degradation performance of 8 wt% CeO_2_/g-C_3_N_4_ (15 mg) toward TC solutions (10–50 mg/L) under photo-Fenton conditions. The degradation efficiency for the high-concentration TC solution (50 mg/L) exhibited reduced efficiency at the given catalyst loading, primarily attributed to the limited surface-active sites of the catalyst, where the photo-Fenton reaction was significantly inhibited. At lower TC concentrations (10 mg/L), the catalyst demonstrated a 56.7% dark adsorption capacity and achieved 83% degradation efficiency after 30 min of irradiation, with a reaction rate constant of 0.04919 min^−1^ (Figure 11d), substantially higher than those observed at higher TC concentrations.

**Figure 12:**
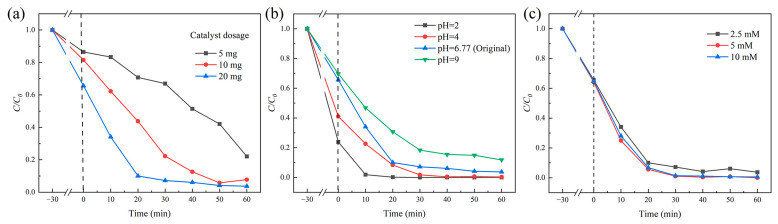
Photo-Fenton degradation of RhB by 8 wt% CeO_2_/g-C_3_N_4_ samples under different experimental conditions; (**a**) catalyst dosage; (**b**) pH value; (**c**) H_2_O_2_ concentration (pollutant concentration: 30 mg/L; light intensity: 100 mW/cm^2^).

The optimization of the photo-Fenton performance for 8 wt% CeO_2_/g-C_3_N_4_ was conducted by systematically investigating the effects of catalyst loading, pH, and H_2_O_2_ concentration on degradation efficiency. As illustrated in Figure 12a, increasing the catalyst loading from 5 mg to 20 mg enhanced the degradation rate from 77.9% to 96.3% at a fixed RhB concentration of 30 mg/L. These results suggest that increases in photoexcited electrons and active sites promote the decomposition of RhB [61]. Additionally, pH significantly influenced the degradation efficiency, with RhB adsorption and photoreduction activities of 8 wt% CeO_2_/g-C_3_N_4_ samples progressively increasing at lower pH values. Figure 12b demonstrates that strong electrostatic attraction between the pollutant and catalyst at pH 2 resulted in a maximum degradation efficiency of 100% [62]. The variation in H_2_O_2_ concentration (Figure 12c) exhibited minimal impact on RhB degradation efficiency, with all conditions achieving approximately 98% degradation.

**Figure 13:**
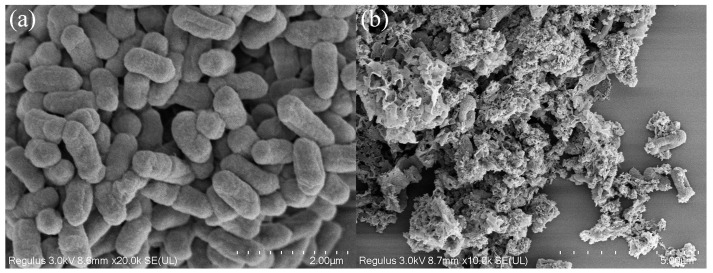
Scanning electron microscopy images of cells before (**a**) and after (**b**) photo-Fenton treatment.

Morphological changes in bacterial cells induced by photo-Fenton treatment were investigated using SEM imaging (Figure 13). The untreated bacterial cells (Figure 13a) displayed characteristic smooth surfaces and intact morphological features. Post-photocatalysis (Figure 13b), cells displayed significant morphological alterations, including membrane concavity, structural perforation, and cytoplasmic leakage. These observations demonstrate that ROS generated during the photo-Fenton process induce substantial cellular damage through the oxidative degradation of cell membranes and walls, leading to structural collapse and cellular death [63]. Wei et al. [64] corroborated these findings, demonstrating that photocatalysts facilitate both membrane disruption and DNA degradation.

### 2.5. Fluorescence Staining Tests

**Figure 14:**
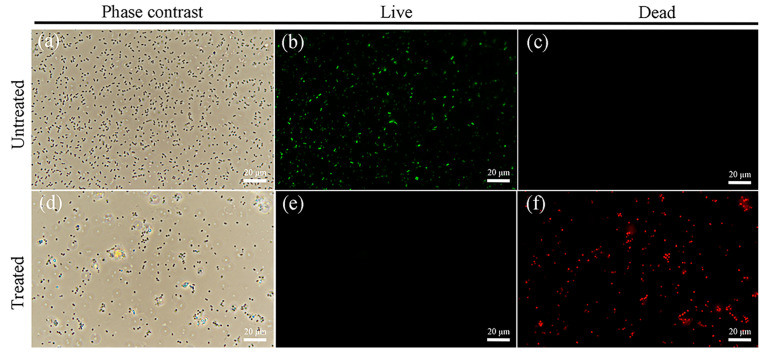
(**a**,**d**) Phase contrast microscope images of the corresponding fluorescent images (**b**,**c**,**e**,**f**) showing the cell viability of *E. coli* post 40 min illumination, (**a**–**c**) without photo-Fenton treatment or (**d**–**f**) with photo-Fenton of 8 wt% CeO_2_/g-C_3_N_4_. Cells were stained with CFDA, AM/Propidium Iodide: the live cells fluoresce green; the dead cells fluoresce (red, dead bacteria; green, live bacteria).

Bacterial viability was assessed using live/dead fluorescence staining analysis, wherein green fluorescence indicates viable cells, and red fluorescence denotes non-viable cells under fluorescent phase-contrast microscopy [65]. Phase-contrast microscopy and fluorescence staining images of untreated cells (Figure 14a–c) revealed predominantly green fluorescence, confirming high cellular viability. Following photo-Fenton treatment, cells predominantly displayed red fluorescence, confirming the significant loss of cellular viability.

### 2.6. Active Oxygen Species Tests

**Figure 15:**
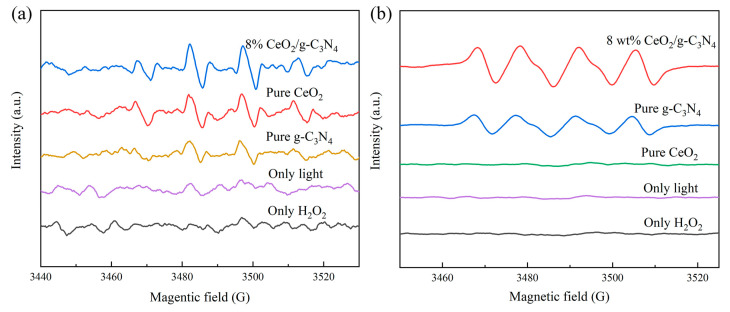
The (**a**) ·OH and (**b**) ·O_2_^−^ signals of different samples.

ESR spectroscopy was employed to investigate the radical species generated by 8 wt% CeO_2_/g-C_3_N_4_ (Figure 15). Under sole illumination or hydrogen peroxide exposure, no radical signals were detected. In contrast, pristine CeO_2_ and g-C_3_N_4_ exhibited weak ·OH signals with significant noise, whereas 8 wt% CeO_2_/g-C_3_N_4_ demonstrated significantly enhanced ·OH and ·O_2_^−^ signals. This enhancement likely results from heterojunction formation between CeO_2_ and g-C_3_N_4_, promoting efficient carrier separation, consistent with the findings of Li [66] and Yan [67]. These results confirm that both ·OH and ·O_2_^−^ species are crucial for the photo-Fenton disinfection activity of 8 wt% CeO_2_/g-C_3_N_4_ composites [68].

**Figure 16:**
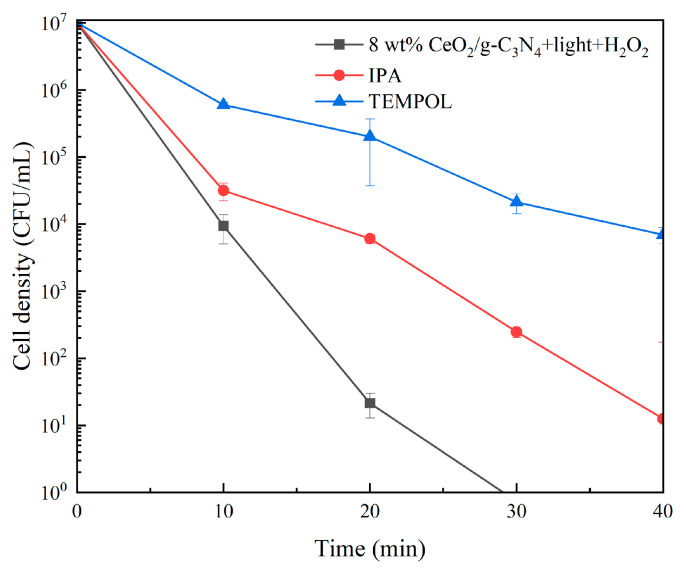
Effect of scavenger on photo-Fenton bacteriostatic rate. (IPA: ·OH; TEMPOL: ·O_2_^−^ ).

The interaction between ROS and cells or pollutant molecules constitutes the primary mechanism of disinfection and degradation processes. To elucidate the dominant ROS in the photo-Fenton process, selective radical scavenging experiments were conducted. As shown in Figure 16, the addition of isopropanol (IPA), a hydroxyl radical scavenger, resulted in a minimal increase in *R. solanacearum* survival. However, the addition of TEMPOL, a superoxide radical scavenger, significantly enhanced bacterial survival, indicating that ·O_2_^−^ plays a predominant role in the photo-Fenton reaction process.

### 2.7. Mammalian Cytotoxicity Tests

**Figure 17:**
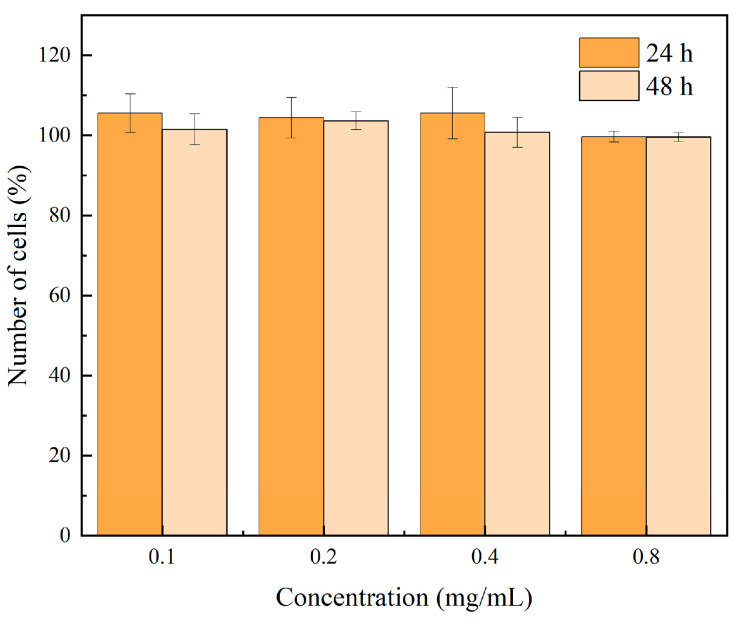
Toxicity of 8 wt% CeO_2_/g-C_3_N_4_ to HEK293 cells.

The assessment of mammalian cell viability serves as one of the key indicators for evaluating toxicological effects. The cytotoxicity of 8 wt% CeO_2_/C_3_N_4_ was evaluated via CCK-8 assay using human embryonic kidney (HEK293) cells. As illustrated in Figure 17, the nanocomposite exhibited negligible cytotoxicity toward HEK293 cells during short-term exposure (12 h), maintaining cell viability at approximately 100%, even after 24 h of incubation at high concentrations (0.8 mg/mL). These results indicate that the 8 wt% CeO_2_/C_3_N_4_ nanocomposite demonstrates excellent biocompatibility and potential applications as an environmentally friendly material.

## 3. Material and Methods

### 3.1. Experimental Reagents

All chemicals were of analytical grade and used as received. The following materials were used: urea (CH_4_N_2_O, 99%, Sinopharm, Beijing, China), thioacetamide (TAA, 99%, Sigma-Aldrich, St. Louis, MO, USA), sodium chloride (NaCl, 99.5%, Tianjin Fengchuan, Tianjin, China), potassium chloride (KCl, 99.5%, Tianjin Fengchuan), potassium dihydrogen phosphate (KH_2_PO_4_, 99%, Tianjin Fengchuan), disodium hydrogen phosphate (Na_2_HPO_4_, 99%, Tianjin Fengchuan), yeast extract (Beijing Aoboxing, Beijing, China), nutrient agar (Beijing Aoboxing), cerium chloride (CeCl_3_·7H_2_O, 99%, Shanghai Macklin, Shanghai, China), sodium hydroxide (NaOH, 99%, Sinopharm), anhydrous ethanol (C_2_H_5_OH, 99.7%, Sinopharm), 5, 5-dimethyl-1-pyrrolin-n-oxide (C_6_H_11_NO, Shanghai Macklin), Nafion ((C_7_HF_13_O_5_S.C_2_F_4_)x, Shanghai Macklin), sodium sulfate (Na_2_SO_4_, Sinopharm), rhodamine B (RhB, Tianjin Kermel, Tianjin, China), tetracycline hydrochloride (TC, McLean Biochemical, Shanghai, China), and hydrogen peroxide (H_2_O_2_, 30%, Shanghai Macklin).

### 3.2. Preparation of CeO_2_/g-C_3_N_4_ Nanocomposites

The synthesis of CeO_2_/g-C_3_N_4_ nanocomposites was performed as follows: 8 g of urea and 4 g of melamine were dispersed in 50 mL of deionized water and homogenized under magnetic stirring to form solution A. Separately, predetermined amounts of CeCl_3_·7H_2_O were dissolved in 50 mL of deionized water to prepare solution B. Solution B was added dropwise to solution A under vigorous stirring. The resulting mixture was concentrated by heating in a water bath at 70 °C, followed by calcination in a muffle furnace at 550 °C for 4 h (heating rate: 5 °C/min). The resulting materials were designated as x% CeO_2_/g-C_3_N_4_, where x = 4, 6, 8, and 10, representing the mass percentage of CeO_2_.

### 3.3. Characterization

Powder X-ray diffraction (XRD) patterns were collected on a D8 Advance diffractometer (Bruker, Karlsruhe, Germany) using Cu Kα radiation. The morphology and microstructure were characterized using transmission electron microscopy (TEM, JEM-2100, JEOL, Akishima, Japan) operated at 200 kV and field-emission scanning electron microscopy (FESEM, JSM-7001F, JEOL, Akishima, Japan). Surface chemical composition and electronic states were investigated by X-ray photoelectron spectroscopy (XPS, ESCALAB 250, Thermo Fisher Scientific, Waltham, MA, USA) with Al Kα radiation. Functional group identification was conducted using Fourier transform infrared spectroscopy (FTIR, Vertex, Bruker, Germany) in the range of 4000–500 cm^−1^. Photophysical properties were characterized using fluorescence spectrophotometry (PL, 930 N, Shanghai, China) with excitation at 365 nm and UV-visible diffuse reflectance spectroscopy (UV-Vis DRS, U-3900H, Hitachi, Tokyo, Japan). Active species generation was monitored using electron spin resonance spectroscopy (ESR, Escan, Bruker, Karlsruhe, Germany), with DMPO as the spin-trapping agent. Photoelectrochemical measurements were performed using a CHI660E electrochemical workstation (CH Instruments, Shanghai, China) in a standard three-electrode configuration, with Ag/AgCl as the reference electrode and Pt wire as the counter electrode.

### 3.4. Photoelectrochemical Property Testing

Photoelectrochemical measurements were performed to evaluate charge separation efficiency and interfacial charge transfer characteristics of CeO_2_/g-C_3_N_4_ nanocomposites. The photocurrent response characterizes charge carrier generation and separation efficiency, where higher photocurrent density corresponds to enhanced charge carrier separation. Additionally, electrochemical impedance spectroscopy (EIS) was performed, where a smaller arc radius in the EIS Nyquist plot indicated reduced charge transfer resistance and improved photocatalytic performance. Working electrodes were prepared by dispersing 5 mg of catalyst powder in 5 mL of absolute ethanol containing 5 μL of Nafion (5 wt%) as a binder. The suspension was sonicated to achieve homogeneous dispersion. The resulting mixture was drop-cast onto fluorine-doped tin oxide (FTO) glass substrates. After the initial layer was completely dried, a second layer was applied following the same procedure to ensure uniform coverage. Measurements were performed in a three-electrode configuration using 0.1 M Na_2_SO_4_ as the electrolyte, with platinum as the counter electrode and Ag/AgCl as the reference electrode. Photoresponse was measured under illumination from a 300 W xenon lamp (light intensity: 30 mW/cm^2^).

### 3.5. Photo-Fenton Sterilization Experiment

The photo-Fenton antimicrobial activity of CeO_2_/g-C_3_N_4_ nanocomposites was evaluated against *E. coli*, *R. solanacearum*, and *S. aureus*. Using *E. coli* as a model organism, the following procedure was implemented: The light source was pre-heated and calibrated to the desired intensity. The photocatalytic system consisted of 10 mg of catalyst dispersed in 9.9 mL of sterile PBS buffer (pH 7.4) in 60 mm × 15 mm Petri dishes via sonication (10 min). Subsequently, 0.1 mL of bacterial suspension (2 × 10^9^ CFU/mL) and 5 mM H_2_O_2_ were added under magnetic stirring. Aliquots (0.1 mL) were collected at 10 min intervals and serially diluted with PBS buffer to achieve concentrations of 2 × 10^3^, 2 × 10^5^, and 2 × 10^7^ CFU/mL. From each dilution, 0.1 mL was spread-plated onto LB agar medium. Plates were incubated inverted at 37 °C for 12–24 h. Viable colonies were enumerated using the standard plate counting method, considering plates with 20–300 colonies as statistically valid. All experiments were performed in triplicate, and results were expressed as the survival ratio (N_t_/N_t0_), where N_t_ represents the colony count at time t and N_t0_ the initial count. Control experiments were conducted without catalyst (light + bacteria) and with H_2_O_2_ only (H_2_O_2_ + bacteria).

### 3.6. Photocatalytic Degradation of RhB and TC

RhB and TC were selected as model organic pollutants, with initial concentrations of 10, 30, and 50 mg/L. The photocatalytic experiments were conducted by dispersing 15 mg of catalyst in 40 mL of pollutant solution. The suspension was maintained under magnetic stirring in dark conditions for 30 min to establish adsorption–desorption equilibrium. Irradiation was provided by a 300 W xenon lamp equipped with a 400 nm cutoff filter. The photo-Fenton process was initiated by adding H_2_O_2_ (5 mM). Aliquots (2 mL) were collected at 10 min intervals and filtered through 0.22 μm membranes to remove suspended particles. The degradation progress was monitored by measuring the filtrate absorbance using UV-visible spectrophotometry at λmax = 554 nm for RhB and 357 nm for TC.

## 4. Conclusions

In this study, two-dimensional CeO_2_/g-C_3_N_4_ nanocomposites were synthesized via a straightforward thermal calcination method using thiourea and urea as precursors. The detailed physicochemical analyses confirmed that the heterojunction structure led to markedly improved light absorption and charge carrier separation efficiency, compared to pristine CeO_2_ and g-C_3_N_4_. Mechanistic studies using ESR spectroscopy and antimicrobial assays established that the composite materials achieved bacterial inactivation through the generation of ·OH and ·O_2_^−^. The optimized 8 wt% CeO_2_/g-C_3_N_4_ exhibited enhanced bactericidal activity, achieving complete inactivation of *E. coli*, *R. solanacearum,* and *S. aureus* (initial concentration: 2 × 10^7^ CFU/mL) within 40 min and 2 h, respectively. These findings not only advance our understanding of g-C_3_N_4_-based heterojunction photocatalysts for efficient photo-Fenton disinfection applications but also demonstrate their practical potential for water treatment applications.

## Data Availability

The data presented in this study are available upon request from the authors.
